# Enabling elliptically polarized high harmonic generation with short cross polarized laser pulses

**DOI:** 10.1038/s41598-023-39814-y

**Published:** 2023-08-08

**Authors:** B. Ghomashi, S. Walker, A. Becker

**Affiliations:** JILA and Department of Physics, University of Colorado, Boulder, CO 80309-0440 USA

**Keywords:** High-harmonic generation, Nonlinear optics

## Abstract

Enabling elliptically polarized high-order harmonics overcomes a historical limitation in the generation of this highly nonlinear process in atomic, molecular and optical physics with applications in other branches. Here, we shed new light on a controversy between experimental observations and theoretical predictions on the possibility to generate harmonics with large ellipticity using two bichromatic laser pulses which are linearly polarized in orthogonal directions. Results of numerical calculations confirm the previous experimental data that in short laser pulses even harmonics with large ellipticity can be obtained for the interaction of such cross-polarized laser pulses with atoms initially in a *s*- or *p*-state, while odd harmonics have low ellipticity. The amount of the ellipticity can be controlled via the relative carrier-envelope phase of the pulses, their intensity ratio and the duration of the pulses.

## Introduction

Extreme ultraviolet (XUV) and soft x-ray radiation are important light tools for studying the dynamics of fundamental processes in atoms, molecules and materials on ultrashort time scales (see recent reviews^1, 2^). High harmonic generation (HHG) provides the opportunity to produce such light in a tabletop experimental setting. It is a highly nonlinear process which results from a gas (or solid) interacting with an intense laser field^3, 4^. On the microscopic (atomic) level the three-step model gives an intuitive picture of the process^5–7^: the intense electric field ionizes an electron from the atom, the electron then propagates in the oscillating field gathering energy, returns to the core and upon recombination with the core emits its energy in an attosecond burst of radiation. In a pulse of several cycles the process repeats every half cycle of the field, giving rise to an attosecond pulse train of high harmonic light. In the frequency domain the light spectrum consists of a series of peaks, or harmonics, at integer multiples of the fundamental frequency reaching up to the soft x-ray regime^8–10^. For a long time experiments generated and applied linearly polarized harmonics, only recently several methods have been proposed and demonstrated to overcome this restriction^11–20^. Since then the ability of generating elliptically polarized ultrashort light pulses in the XUV and soft x-ray wavelength regime has enabled various applications with respect to spin-orbit interaction^21^, molecular symmetries and chirality^22, 23^, circular dichroism^12, 24^, and magnetic interactions^25^.

Following an initial proposal in 1995^26^, most of the techniques for the generation of elliptically polarized high harmonics are based on the application of bichromatic, typically $\omega - 2\omega$, fields in various focusing configurations^11–20^. Perhaps, one of the simplest configurations to implement is the use of two linearly polarized laser pulses at frequencies $\omega$ and $2\omega$ in a geometry in which the polarization vectors are orthogonal to each other. Yet, generation of high harmonics in this configuration entails an important open question. Due to the reflection symmetry in this configuration, it has been predicted that only linearly polarized harmonics are generated, independent of whether the active valence electron in the target atom is initially in a *s*- or in a *p*-state^27, 28^. For the interpretation it is assumed that the driving field is periodic, envelope effects are negligible and that the pulse is long. The analysis then shows that all the odd harmonics will be polarized along the axis parallel to the fundamental field and the even harmonics are polarized along the perpendicular axis. Even relaxing the long-pulse assumption, it was considered that as long as the active orbital is spherically symmetric the generated harmonics can only be linearly polarized in the direction along the recollision angle of the electron trajectory associated with that harmonic. However, in 2015 an experimental study reported the generation of bright highly elliptically polarized high harmonics with cross-polarized bichromatic laser pulses^16^, most prominent at and beyond the HHG cut-off. Very few theoretical studies have addressed this controversy between experimental observation and theoretical prediction so far^29–31^. For example, it has been shown that the assumption that each of the attosecond bursts of high harmonic generation is linearly polarized^27, 28^ is inaccurate^32^. However, an overarching picture explaining the experimental observations has not emerged to the best of our knowledge.

With this work we intend to provide new insights into the feasibility of generating elliptically polarized high harmonics at and beyond the cut-off with cross-polarized bichromatic laser pulses. Using ab-initio microscopic solutions of the time-dependent Schrödinger equation we show that indeed highly elliptically polarized harmonics from a *s*-valence orbital in atomic hydrogen and from a *p*-orbital in atomic argon can be generated. This confirms the experimental observations, including the result that the ellipticities of the generated even harmonics are in general larger than those of the odd harmonics. We relate the observed ellipticity to the parameters of the fields, which provides an intuitive picture of the underlying physical process, and address differences in the results depending on the initial orbital shape. Finally, we show that restricting the effective interaction time of both pulses, e.g. by shortening the pulses, is essential for achieving high ellipticities.

## Results and discussion

**Figure 1:**
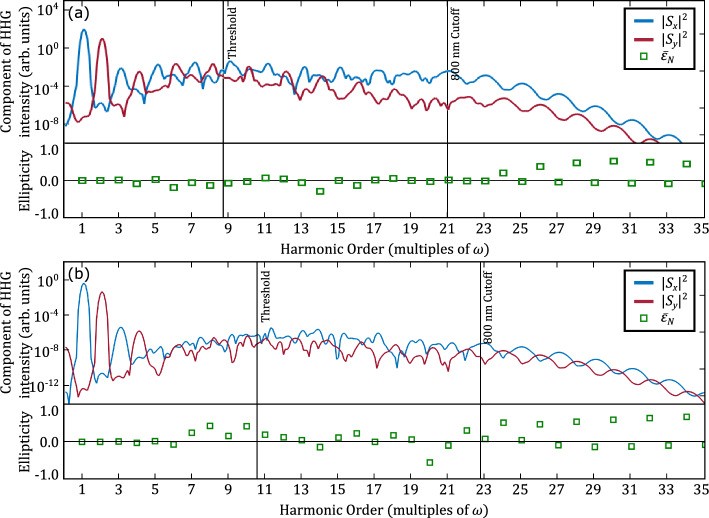
Components of HHG spectra and ellipticity for the interaction of (**a**) a hydrogen atom and (**b**) an argon atom with an intense laser pulse. In both cases the driving laser intensities are $I_{800} = 10^{14}$ W/$\hbox {cm}^2$ and $I_{400} = 10^{13}$ W/$\hbox {cm}^2$ with a duration of 26.8 fs, which corresponds to 10 (20) cycles of the fundamental ($2\omega$) field. The carrier envelope phase (CEP) of the 800 nm component is $\phi _{\textrm{800}}=0$ while the CEP of the 400 nm component is $\phi ^{(\mathrm H)}_{\textrm{400}}=-\frac{\pi }{4}$ for hydrogen and $\phi ^{(\mathrm Ar)}_{\textrm{400}}=-\frac{5\pi }{8}$ for argon. Solid vertical lines indicate the ionization threshold and the cutoff due to the stronger 800 nm pulse.

In Fig. 1 we display the results of numerical calculations, based on the solution of the time-dependent Schrödinger equation (see “Methods”), for microscopic high-harmonic spectra obtained for the interaction of (a) a hydrogen atom and (b) an argon atom with cross-polarized laser pulses. For the argon atom the calculations were performed in the single-active electron approximation, using a single-active-electron potential fitted to a density functional theory calculation^33^. The two laser pulses were centered at 800 nm ($\omega$-field, polarized in ${\hat{\textbf{x}}}$-direction) and at 400 nm ($2\omega$-field, polarized in ${\hat{\textbf{y}}}$-direction). Like in the experiment^16^, we used a high peak intensity $I_{\omega } = 10^{14}$ W/$\hbox {cm}^2$ for the field at the fundamental wavelength, while the second harmonic pulse intensity was an order of magnitude lower, $I_{2\omega } = 10^{13}$ W/$\hbox {cm}^2$. Both pulses had the same duration with the peak of the envelopes coinciding and the relative carrier-to-envelope phases as given in the Figure caption.

Each panel of the Fig. 1 consists of two graphs: in the upper graph shown are the intensities of the components of the generated high harmonic fields in the ${\hat{\textbf{x}}}$-direction (blue line) and in the ${\hat{\textbf{y}}}$-direction (red line) as a function of multiples of the fundamental frequency. According to our calculations and in agreement with theoretical predictions the component in the direction perpendicular to the polarization plane (i.e., the ${\hat{\textbf{z}}}$-direction) is negligible and therefore not shown. As it is expected, the HHG intensities in the polarization direction (i.e., ${\hat{\textbf{x}}}$-direction) of the fundamental driving field, display maxima at odd harmonics, while those in the orthogonal direction have maxima at the even harmonics. This trend is most clearly visible for harmonics below the ionization threshold and near and past the cut-off, which are indicated by the vertical lines in the Figure.

We have also obtained the ellipticities of the generated harmonics (open squares), by defining the ellipticity at a given frequency $\Omega$ as^32^:

1 $$\begin{aligned} \varepsilon (\Omega ) =-\tan \left[ \frac{1}{2}\arcsin \left( \frac{2\rho (\Omega )}{1 + \rho ^2(\Omega )}\sin (\phi _y(\Omega )-\phi _x(\Omega )) \right) \right] , \end{aligned}$$

where $\rho (\Omega )=\left| S_y(\Omega )/S_x(\Omega )\right|$ and $S_{x,y}$ are the amplitudes of the harmonics in the two orthogonal directions. We then obtained the ellipticity at a given harmonic order as an average over the range of one harmonic using a Gaussian filter:

2 $$\begin{aligned} {\bar{\varepsilon }}_N=\frac{\int _{-\infty }^\infty \varepsilon (\Omega ) |{\textbf{S}}(\Omega )|^2e^{-\frac{1}{2\sigma ^2}\left( \frac{\Omega }{\omega }-N\right) ^2}d\Omega }{\int _{-\infty }^\infty |{\textbf{S}}(\Omega )|^2e^{-\frac{1}{2\sigma ^2}\left( \frac{\Omega }{\omega }-N\right) ^2}d\Omega }, \end{aligned}$$

where $\sigma =0.125$ is a dimensionless quantity. The results show that the even harmonics near, at and past the cut off have a significant amount of ellipticity while the odd harmonics are in general close to being linearly polarized. This result is in qualitative agreement with the experimental observations^16^. From Eq. (1) we notice that a large ellipticity can be obtained by matching the phase difference of the two components to $\pi /2$ and the ratio of the two components, $\rho (\Omega )$, close to 1. As we can see from the results in Fig. 1 the signal at each of the harmonics is broad and the minima for the *x*-component match up rather well with the maxima of the *y*-components of the generated radiation at the frequencies of the even harmonics with high ellipticity, especially near and at the cut-off. In contrast, we observe a large difference between the respective maxima (in $|S_x|^2$) and minima (in $|S_y|^2$) at the frequencies of the odd harmonics and, hence, those harmonics are close to being linearly polarized. Indeed, we can generalize this observation: If the intensity of the second harmonic field is sufficiently weaker than that of the fundamental pulse, we can expect that the component of the high harmonic generation in the direction of the $2\omega$-field is weaker than the component in the direction of the $\omega$-field. Therefore, the requirement of matching the intensities of the two components for generating high-order harmonics with a high ellipticity is more likely to be achieved for the even than for the odd harmonics. In order to address these points further, we will discuss in the remainder of this work which parameters of the two fields impact the degree of ellipticity.

**Figure 2:**
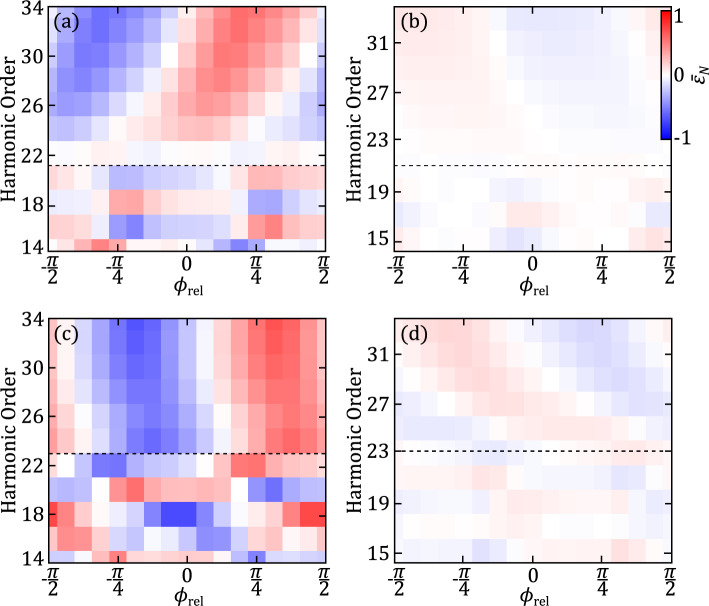
Ellipticity of the even (panels on the left) and odd (panels on the right) harmonics as a function of harmonic order and relative phase $\phi _{\textrm{rel}} = \phi _{800}-\frac{\phi _{400}}{2}$ for (**a**, **b**) hydrogen atom and (**c**, **d**) argon atom. The dashed line indicates the harmonic cut-off, associated with the stronger 800 nm field. Other laser parameters are the same as in Fig. 1.

First, we show in Fig. 2 results of additional calculations for hydrogen (panels in upper row) and argon (panels in lower row) in which the carrier waves of the two cross-polarized fields are offset to each other while the envelope remains unchanged. The relative phase of the two fields, $\phi _{\textrm{rel}} = \phi _{800}-\phi _{400}/2$, is varied over one cycle of the fundamental field. Shown are the ellipticities of the (a) even and (b) odd harmonics as function of the relative phase of the $2\omega$-field measured with respect to the peak of the fundamental field. The results show a few distinct features: First, large values of ellipticity are found only for even harmonics, while the odd harmonics are close to linearly polarized independent of the relative phase of the two fields. Next, the ellipticity for the even harmonics varies largely as a function of the relative phase. While for the harmonics near and beyond the cut-off one sees a smooth variation with two extrema having opposite signs, there is no order in the pattern of the plateau harmonics. This indicates that the generation of elliptically polarized harmonics around and after the cut-off can be more easily observed and controlled which may explain why in the experimental report^16^ ellipticities for cut-off harmonics have been reported. Furthermore, we expect that the inherent physical picture behind the results may be more evident at the highest harmonics. Within the three-step model of high harmonic generation ionization and recombination times for electrons leading to the generation of the harmonics can be identified. According to this picture the relative phase, at which maximum values of ellipticities for the cut-off harmonics are found, indicates that the maximum of the $2\omega$-field oscillation has to occur slightly after the respective ionization time.

**Figure 3:**
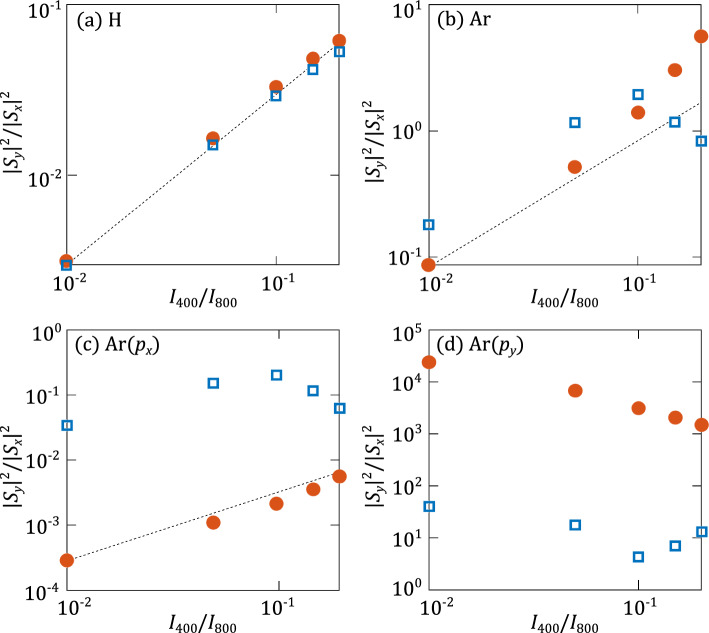
Ratio of radiation polarized in the $\hat{\textbf{y}}$-direction to radiation polarized in the $\hat{\textbf{x}}$-direction emitted from (**a**) hydrogen atom and (**b**) argon atom as a function of the intensity ratio of the two fields. The intensity of the fundamental field was kept fixed at $10^{14}$ W/$\hbox {cm}^2$, while that of the $2\omega$-field was varied. Separately shown are the results from the (**c**) $p_x$- and (**d**) $p_y$-orbital in argon. Blue squares represent the results for a plateau harmonic (harmonic 18) and red squares those for a cut-off harmonic (harmonic 24). The dotted lines indicate a linear increase and are shown for the sake of comparison. Other laser parameters are the same as in Fig. 1.

While it is more difficult to control the phase difference between the *x*- and *y*-components of the harmonic radiation, the relative strength of the components is expected to depend on the intensity ratio of the two cross polarized fields, especially when the intensity of the 400 nm field is significantly smaller than the intensity of the 800 nm field. To confirm this expectation, we changed the intensity ratio of the fields by keeping the intensity of the fundamental pulse at $10^{14}$ W/$\hbox {cm}^2$ while varying the intensity of the second harmonic field. As examples of the results we show the ratio of the radiation polarized in the $\hat{\textbf{y}}$-direction to that polarized in the $\hat{\textbf{x}}$-direction, i.e. $\rho ^2(\Omega )$, for an even harmonic in the plateau (harmonic 18, blue squares) and in the cut-off region (harmonic 24, red circles) in Fig. 3. For hydrogen atom (panel a) we see that the harmonic intensity ratio increases linearly with respect to the 400 nm field intensity. This observation implies that the ratio, more specifically the $S_y$-component, depends on the interaction with just a single photon from the weaker $2\omega$-field. The slight deviation from the linear trend for the plateau harmonic (harmonic 18, blue squares) at the highest field intensity ratios likely indicates the impact of higher order 400 nm photon processes. We observe that the results for the $p_x$-orbital in argon atom (panel c) are quite similar to those found for the hydrogen atom. This can be understood since the orientation of the orbital is along the polarization direction of the stronger fundamental field and, hence, the coupling with the 800 nm photons is much stronger than with the 400 nm field, which is polarized in the orthogonal direction. In contrast, for the $p_y$-orbital, oriented in the direction of the weaker $2\omega$-field, the results are different. The $S_y$-component is much stronger than the $S_x$-component and the overall trend of the harmonic intensity ratio is decreasing rather than increasing linearly. This means that here we cannot interpret the interaction of the $2\omega$-field as a single photon coupling or being perturbative with respect to the interaction with the 800 nm field. For the full Ar results (panel b) we therefore conclude that the linear trend at the lowest field intensity ratios is likely due to the dominant impact from the contributions of the harmonic radiation from the $p_x$-orbital, while at higher ratios the contribution from the $p_y$-orbital becomes more significant, leading to the deviation from the linear increase of the harmonic intensity ratio. We may note that indication of the (first order) perturbative impact of the second harmonic field can also be seen in the results presented in Fig. 2. Beyond the cut-off the ellipticity of the even harmonics (panels on the left) varies periodically as a function of the relative phase (or, equivalently, as a function of the carrier-envelope phase of the 400 nm field), indicating the impact of a single photon transition by the weaker second harmonic field. In contrast, the variation is more complex for harmonics before the cut-off. Furthermore, the observation that highest ellipticities are found when the maximum of the $2\omega$-field occurs slightly after the respective ionization time driven by the fundamental field may be interpreted as another indication of the weak perturbative impact of the second harmonic field.

**Figure 4:**
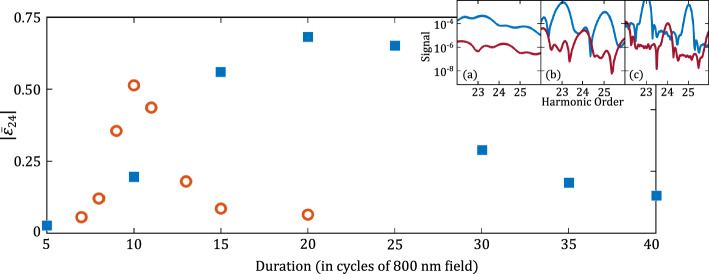
Ellipticity of the 24th harmonic from hydrogen atom (blue squares) and argon atom (red circles) as a function of the duration of the laser pulses (in cycles of the fundamental field). Other laser parameters are the same as in Fig. 1. The insets show the *x*- (blue lines) and the *y*-components of the harmonic signal from hydrogen atom at three pulse durations, (**a**) 7 cycles, (**b**) 20 cycles, and (**c**) 40 cycles.

As mentioned above, an integral part to enable the generation of elliptically polarized high harmonics is to achieve comparable signal components along the polarization directions of the two laser pulses, in particular a match of the minima in the spectral component of the stronger (fundamental) field and the maxima in the spectral component of the weaker (second harmonic) field. Besides controlling the intensity ratio of the two pulses, this means that the duration over which the two pulses interact with the atom has to be kept short. For a short interaction times the harmonic lines are rather broad, limiting the contrast between maxima and minima in each harmonic component, while for long interaction times the harmonic lines tend to get narrower enhancing the difference between the extrema in the spectra. This is shown in the insets of Fig. 4, which show the harmonic components (blue: *x*-component, red: *y*-component) of the 24th harmonic from hydrogen atom. In the left panel the laser pulse duration is very short, the lines are broad, the two components are well separated, and, hence, the polarization of the harmonic is almost linear. At a duration of 20 optical cycles (middle panel) the lines are narrower and more pronounced. This leads to an increase of the maxima and a decrease of the minima in the two components. At this intensity ratio and this duration the two components overlap well for the 24th harmonic, leading to the high ellipticity. At the longest pulse duration considered (right panel) the maximum of the *y*-component and the minimum of the *x*-component are even more pronounced, therefore the overlap of the components for the 24th harmonic is less than at intermediate pulse duration and, thus, the ellipticity of the harmonic is lower. The main results in Fig. 4 confirm the expectation that for each of the atoms the ellipticity of the even harmonics can be enhanced significantly by finding the optimum pulse duration. Indeed, we see that very high ellipticities can be obtained, which is in agreement with the experimental observations^16^. We note that the effective joint interaction time of the two pulses can also be controlled by keeping the pulse duration constant but delaying the pulses to each other and that the optimal pulse duration will depend, at least, on the intensity ratio of the two pulses, and the kind of target atom. These two factors impact the relative strength of the two components of the harmonic signal, which is relevant for the ellipticity of the harmonics.

## Conclusion

In summary, by presenting and interpreting results of numerical calculations for high-harmonic spectra in hydrogen and argon atoms interacting with cross-polarized laser pulses we shed new light on a controversy between experimental observations and theoretical predictions concerning the generation of elliptically polarized high harmonics in this set-up. Our results confirm the experimental observations that, for a short laser pulse, near the cut-off large ellipticities for the even harmonics are generated while those for the odd harmonics remain small. In the long pulse limit our results agree with theoretical predictions that the harmonics are linearly polarized for a periodic cross-polarized field. The analysis of the results provides a picture in which the second harmonic acts as a perturbation after the time of birth of the electron in the continuum by the fundamental field. Furthermore, it is shown that the amount of ellipticity can be controlled via the relative phase between the two pulses, their intensity ratio and the pulse durations. Enabling the generation of elliptically polarized harmonics in this rather simple set-up extends the pool of techniques for helicity-dependent high harmonic generation which have a large application range in dynamical studies on ultrashort time scales.

## Methods

### Single-active electron calculations of high harmonic generation

The theoretical and numerical methods used to obtain the HHG spectra are based on numerical solution of the time-dependent Schrödinger equation. The Hamiltonian for a single-active electron atom in an external electric field is given by (in velocity gauge and dipole approximation, Hartree atomic units are used, $e = m = \hslash = 1$),

3 $$\begin{aligned} H=\frac{{\textbf{p}}^2}{2}+{\textbf{p}}\cdot {\textbf{A}}(t)+V(r), \end{aligned}$$

where ${\textbf{p}}$ is the kinetic momentum operator of the electron, ${\textbf{A}}(t)$ is the vector potential, and *V*(*r*) is the single-active electron atomic potential, which is fitted to a density functional theory calculation^33^. The vector potential of the orthogonal two-color field is

4 $$\begin{aligned} {\textbf{A}}(t)=-A_{800}\sin ^2\left( \frac{\omega t}{2N}\right) \sin (\omega t + \phi _{800}){\hat{x}}- A_{400}\sin ^2\left( \frac{\omega t}{2N}\right) \sin (2\omega t + \phi _{400}){\hat{y}}, \end{aligned}$$

where $\omega$ is the central frequency of the fundamental field, *N* is the number of cycles of the fundamental field, $\phi _{800/400}$ is the carrier envelope phase, $A_{800/400}$ is the amplitude of the *x*/*y* component.

For the numerical calculations the wavefunction is expanded in a basis of spherical harmonics for the angular dimensions and 8th order *B*-splines in the radial dimension. This approach follows the strategy as outlined in^34, 35^. The 300 *B*-splines nodes are placed such that the spacing between nodes is quadratic near the origin then becomes constant at a chosen radius (here, 30 a.u.). Both the maximum orbital angular momentum and maximum magnetic quantum number are fixed to 30. The wavefunction is propagated in a box of 300 a.u. in size with exterior complex scaling being applied to the last 30 a.u., where the radial coordinate is rotated into the complex plane by an angle $\eta =\pi /4$, to avoid reflections off the walls^36^. The wavefunction is propagated in time with the Crank-Nicolson method using a time step of 0.1 a.u. The high harmonic signal, ${\textbf{S}}(\omega )$, is calculated by Fourier transforming the dipole acceleration ${\textbf{a}}(t)$,

$$\begin{aligned} {\textbf{S}}(\omega )=\int _{-\infty }^\infty {\textbf{a}}(t)e^{-i\omega t}dt\,, \end{aligned}$$

which is evaluated using the Ehrenfest’s theorem,

$$\begin{aligned} {\textbf{a}}(t)=\frac{d}{dt}\langle {\textbf{v}}(t)\rangle = -\nabla V({\textbf{r}}). \end{aligned}$$

## Data Availability

The datasets used and/or analysed during the current study are available from the corresponding author on reasonable request.
